# Toxicometabolomic profiling of resistant and susceptible western corn rootworm larvae feeding on Bt maize seedlings

**DOI:** 10.1038/s41598-022-15895-z

**Published:** 2022-07-08

**Authors:** Man P. Huynh, Bruce E. Hibbard, Khanh-Van Ho, Kent S. Shelby

**Affiliations:** 1 Division of Plant Science and Technology, University of Missouri, Columbia, MO USA; 2 Department of Plant Protection, Can Tho University, Can Tho, Vietnam; 3 Plant Genetics Research Unit, USDA-Agricultural Research Service, Columbia, MO USA; 4 Department of Chemistry, University of Missouri, Columbia, MO USA; 5 Molecular Imaging and Theranostics Center, University of Missouri, Columbia, MO USA; 6 Department of Food Technology, Can Tho University, Can Tho, Vietnam; 7 Biological Control of Insects Research Laboratory, USDA-Agricultural Research Service, Columbia, MO USA

**Keywords:** Entomology, Agricultural genetics

## Abstract

The western corn rootworm (WCR), *Diabrotica virgifera virgifera* LeConte, is the most serious pest of maize (*Zea mays* L.) in the U.S. Corn Belt and parts of Europe. Transgenic maize hybrids expressing at least one of the four currently available insecticidal toxins from *Bacillus thuringiensis* (Bt) Berliner, currently the most widely adopted control method in continuous maize, have faltered due to the emergence of resistance. The resistance mechanisms of WCR to Bt toxins are not fully understood. We identified metabolic profiles of susceptible and resistant WCR larvae fed on maize hybrids expressing each of three available Cry3 proteins (eCry3Ab1, mCry3A, and Cry3Bb1) targeting corn rootworms and a control non-Bt maize via an untargeted metabolomics approach. Over 580 unique metabolites found in WCR larvae were classified into different pathways (amino acids, carbohydrates, cofactors and vitamins, energy, lipid, nucleotide, peptide, and xenobiotics). By exploring shifts in WCR larval metabolome exclusively by Bt toxins, several candidate metabolites and metabolic pathways were identified in susceptible and resistant larvae that may be involved in defense against or recovery from Bt ingestion by these larvae. These findings would provide mechanistic insights into altered metabolic pathways associated with the resistance mechanisms of WCR to Bt toxins.

## Introduction

The western corn rootworm (WCR), *Diabrotica virgifera virgifera* LeConte (Coleoptera: Chrysomelidae), is an economically important pest of maize (*Zea mays* L.) in the United States and parts of Europe, costing approximately 2 billion dollars (USD) annually in economic losses stemming from yield reductions and control expenses^1^. Larval feeding on maize roots is responsible for the majority of damage associated with this pest, while adult feeding on silks, pollen, kernels and foliage can occasionally cause yield loss if present at high density before anthesis^2^. Historically, the management of WCR relied on chemical insecticides and cultural control techniques such as crop rotation^3,4^. These control tactics have faltered in some areas due to the adaptation of this pest^5–7^. Transgenic maize hybrids expressing at least one of the four currently available insecticidal toxins from *Bacillus thuringiensis* (Bt) Berliner are currently the most widely adopted control method in continuous maize. This control method was first available in 2003 by the commercialization of Bt maize expressing Cry3Bb1 protein, followed by the addition of three Bt toxins Cry34Ab1/Cry35Ab1 [reassigned as Gpp34Ab1/Tpp35Ab1^8^], mCry3A, and eCry3.1Ab, respectively^9^. WCR has been reported to have evolved resistance to Bt maize^10–14^ with cross-resistance between three of the four available Bt toxins^12,15,16^.

Understanding of resistance mechanisms to Bt toxins would be a crucial step in mitigating resistance development in insects. Current insights in Bt mode(s) of action reveal that Cry entomotoxic proteins are δ pore-forming proteins which disrupt the midgut epithelium, leading to the death of the insect host by septicemia^17^. Upon ingestion by an insect, the Cry proteins are solubilized by the host gut fluids and bind to specific receptors on the luminal midgut brush border membrane epithelium^18^. Several functional receptors have been found in the binding sites located in diverse proteins and glycoconjugates^19^ for Cry toxins, including cadherin-like, aminopeptidase-*N* (APN), alkaline phosphatase (ALP), and ATP-binding cassette (ABC) transporter family proteins^20^. The insertion of the Cry protein oligomer into the enterocyte membrane forms an ion pore channel, resulting in an osmotic imbalance due to an influx of extracellular Ca^2+^ and cell death caused by compensatory water influx through aquaporins^21^. The death of enterocytes breaches the midgut epithelial barrier resulting in leakage of gut contents allowing an invasion of the hemocoel by Bt and other gut-resident bacteria, eventually causing the death of the insect by septicemia^22^. Another proposed mode of action is that the binding of Cry proteins to cadherin directly triggers an intracellular adenylate cyclase signaling cascade that results in an activation of protein kinase A and cell death (apoptosis)^23^. Additional contributors to Bt resistance may be the insect microbiome^24,25^. We observed that WCR selected for eCry3.1Ab resistance over many generations possessed a larval microbiome less rich and distinct from the unselected control line^26^. Indeed, simply feeding on eCry3.1Ab maize seedlings caused a rapid shift in larval WCR microbiome composition. Changes in microbiomes, and their associated metabolites could conceivably interact with any of the mechanisms proposed above.

Most of the findings on the resistance mechanisms in insects to Bt toxins are obtained from studies on Bt resistant species of Lepidoptera. Currently, limited information is available on mechanistic data for resistance to Bt toxins in the Coleoptera^17^. In WCR, the binding of mCry3A to brush border membrane vesicles of midgut tissue was reduced in resistant larvae when compared to that of susceptible larvae^27^. An ABC subfamily B transporter protein (ABCB1) was found as a functional receptor of Cry3Aa in WCR. Heterologous expression of this gene in Sf9 and HEK293 cells conferred sensitivity to the cytotoxic effects of the Cry3Aa toxin^28^. Further knockdown of ABCB1 by RNA interference rendered WCR larvae insensitive to the Cry3Aa toxin^28^. This protein was previously identified as a functional receptor for Cry3Aa in another coleopteran species, the aspen leaf beetle, *Chrysomela tremula* Fabricius^29^; however, ABCB1 was not a functional receptor for multiple insecticidal proteins against WCR including Gpp34Ab1/Tpp35Ab1, Cry6Aa1, and IPD072Aa^28^. Additionally, cadherin has been documented to not be a functional receptor of Cry3 toxins in WCR, though this protein has been reported as one of the binding sites of Cry3Bb1 in WCR^30^. Knockdown of cadherin transcripts by RNAi did not affect the susceptibility of WCR to Cry3Aa or Gpp34/Tpp35Ab1^31^. Results from gene expression analyses showed no significant difference in the expression of cadherin between susceptible and resistant larvae exposed to Cry3Bb1 or eCry3.1Ab^32,33^. Compared to susceptible larvae, eCry3.1Ab-resistant larvae exhibited upregulation of esterase and dynein that may be associated with detoxification and midgut repair processes in resistant rootworms^33^.

The metabolome, as a snapshot of carbon flux within an organism, is the most proximal manifestation of a changing phenotype resulting from environmental stressors such as entomotoxins^34^. An untargeted metabolomics analysis provides comprehensive measurement of the entire metabolome^35^, which may be used in an exploratory manner to determine resistance mechanisms, followed by targeted analyses defined from the untargeted metabolomics approach to confirm the resistance mechanism. One recent application of toxicometabolomics was an effort to identify the function of a mutant ABC transporter (ATP Binding Cassette subfamily C2, or SfABCC2) that confers resistance to the Bt entomotoxin Cry1F on fall armyworm larvae^36^. The wild-type SfABCC2 is postulated to serve as a ligand for Cry1F binding on the midgut epithelium. SfABCC2 was assumed to have the normal function of this superfamily of proteins, i.e., ATP-dependent pumping of metabolites (allocrites) from cells. In an attempt to assign function to this midgut ABC transporter, Abdelgaffar et al.^37^ examined the differences in midgut luminal metabolites between resistant and susceptible fall armyworm larvae feeding on maize and artificial diets, hypothesizing that the mutant SfABCC2 transporter would also be an inactive transporter, leading to less allocrite pumped into the lumen. They were able to identify several candidate allocrites for later confirmation^37^. In WCR, the application of a metabolomics approach to examine maize root metabolomes in combination with plant genetics and insect RNAi led to the identification of nonvolatile cues (sugars and benzoxazinoids) in maize roots driving host finding and acceptance of this pest^38^. Nonetheless, the WCR metabolome has never been reported in the refereed literature. Exploration of the shifts in WCR metabolomes caused by the consumption of Bt toxins could provide insights into the resistance mechanisms of WCR to Bt maize.

In the current study, we performed the first untargeted metabolomics analysis to determine metabolic profiles of susceptible and resistant WCR larvae fed on maize hybrids expressing each of three available Cry3 proteins (eCry3Ab1, mCry3A, and Cry3Bb1) targeting corn rootworms and a non-Bt maize. These profiles were compared to identify metabolites that may be involved in the defense against, or recovery from, Bt intoxication by both susceptible and resistant insects. Subsequently, the pathways in which the metabolites expressed differently in the susceptible and resistant larvae fed on maize hybrids with and without the Cry3 proteins were identified.

## Results

### Toxicometabolomics of western corn rootworm larvae

Global metabolomic analysis of whole WCR larvae that fed on maize with and without expressing Bt toxins resulted in the identification of 724 metabolites including 44.9% and 55.1% of the chemical compounds detected in negative and positive ionization mode, respectively using the Metabolon platform (Tables 1, 2). In the metabolite dataset, 81.4% of the metabolites (589/724 compounds) were confirmed with their authentic standards. The remaining metabolites (18.6%) were putatively annotated based on their available identities (e.g., *m/z*, mass spectra). Within the metabolite dataset, 71.8% of the metabolites matched with the Human Metabolome Database (HMDB). The identified metabolites were classified into different metabolic pathways that included amino acids (26.1%), carbohydrates (6.1%), cofactors and vitamins (6.1%), energy (1.2%), lipids (39.2%), nucleotides (10.4%), peptides (4.6%), and xenobiotics (6.3%).

**Table 1:** Experimental design used to rear resistant and susceptible western corn rootworm larval strains on three Bt-expressing maize lines and one control maize line without Bt.

WCR strains	Maize lines			
	Non-Bt maize	eCry3.1Ab-expressing maize	mCry3A-expressing maize	Cry3Bb1-expressing maize
eCry3.1Ab -resistant	×	×		
mCry3A-resistant	×		×	
Cry3Bb1-resistant	×			×
Susceptible	×	×	×	×

**Table 2:** Classification of metabolites identified in western corn rootworm larvae fed on Bt-expressing maize lines compared to feeding on the non-Bt maize control line.

Pathway	Negative ionization mode	Positive ionization mode	Total
Amino acids	66	123	189
Carbohydrates	38	6	44
Cofactors and vitamins	17	27	44
Energy	8	1	9
Lipid	108	176	284
Nucleotide	46	29	75
Peptide	10	23	33
Xenobiotics	32	14	46
	325	399	724

### Comparative metabolite profiling in a susceptible strain fed on three Bt maize lines and a non-Bt maize line

**Figure 1:**
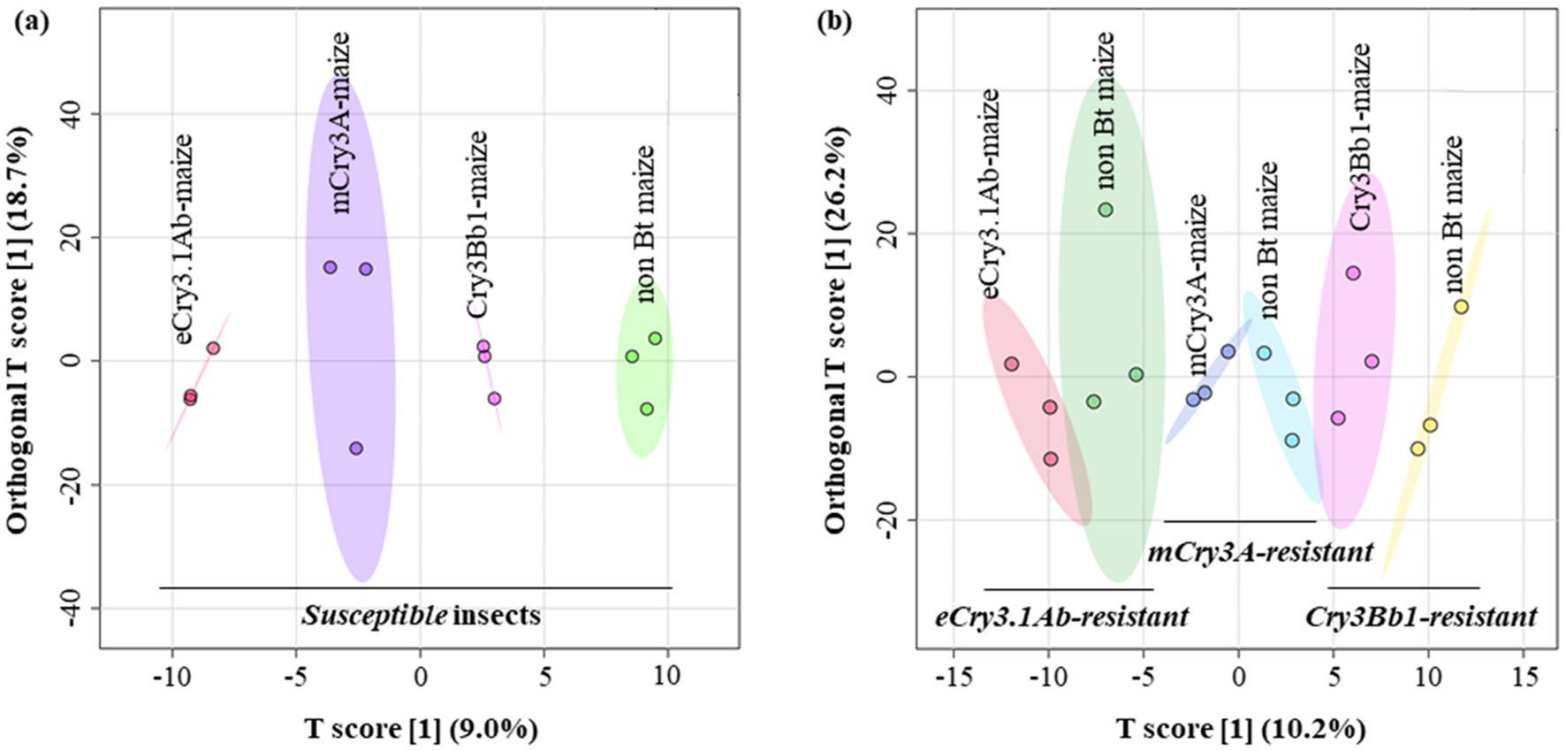
Differences in metabolic profiles of WCR susceptible (**a**) and resistant insects (**b**) fed on control non-Bt maize compared with those fed on eCry3.1Ab-, mCry3A-, or Cry3Bb1-expressing maize seedlings using the orthogonal partial least squares—discriminant analysis (OPLS-DA). In the OPLS-DA plot, circles with same colors represent replicates of metabolic profiles for each treatment. The colored ellipses indicate 95% confidence regions of metabolic profiles for each treatment. In (**a**), from left to right, the circles display susceptible insects fed on eCry3.1Ab-, mCry3A-, Cry3Bb1-expressing maize and non-Bt maize, respectively. In (**b**), from left to right, the circles represent eCry3.1Ab resistant insects fed on eCry3.1Ab-expressing maize and non-Bt maize, mCry3A-resistant insects fed on mCry3A-expressing maize and non-Bt maize, Cry3Bb1-resistant insects fed on Cry3Bb1-expressing maize and non-Bt maize, respectively.

In total, 584 metabolites (~ 81% of the identified metabolite data set) were detected in all samples of susceptible larvae fed on all four examined maize lines (Supplementary Table 1). To characterize differences in metabolite profiles between the susceptible larvae fed on maize hybrids expressing each of the three Bt proteins (eCry3.1Ab, mCry3A, and Cry3Bb1) and those fed a non-Bt maize, orthogonal partial least squares—discriminant analysis (OPLS-DA) was performed. In the OPLS-DA plot, the profiles of the susceptible insects were distributed separately along the T score [1] component but shared a similar pattern regarding the orthogonal T score [1] component (Fig. 1a).

**Figure 2:**
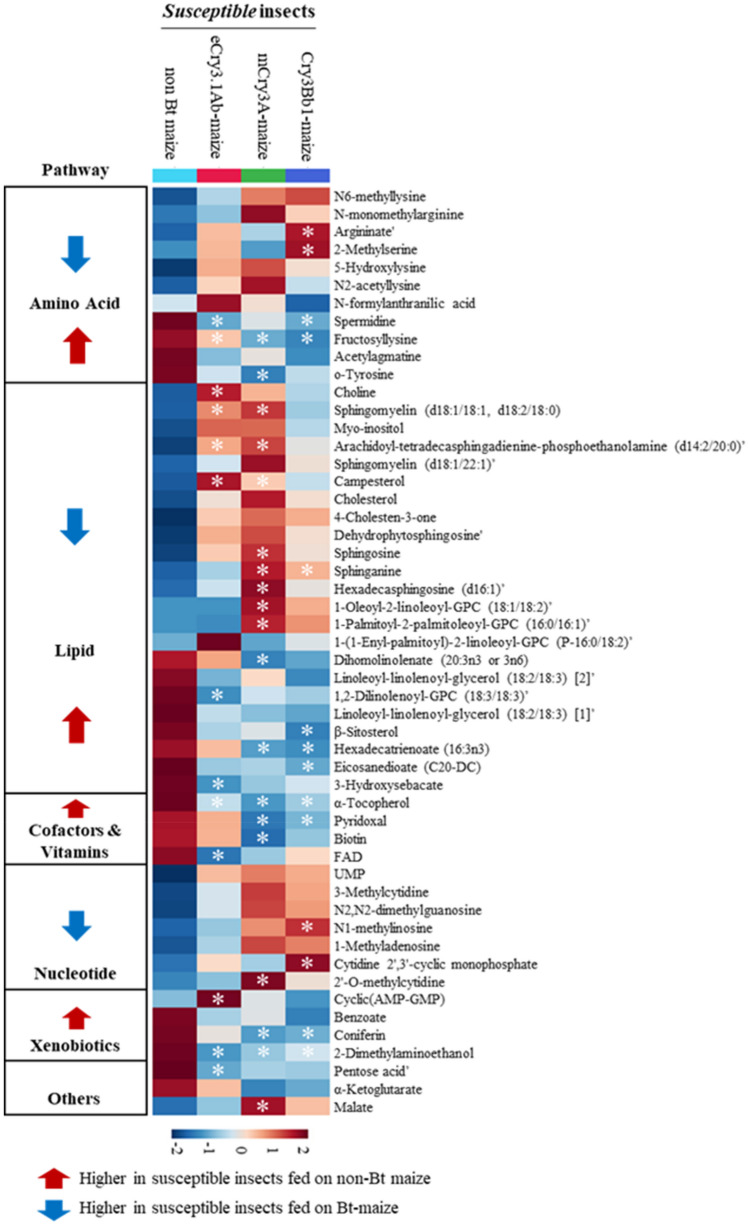
Heatmap representing differences in the levels of identified metabolites expressed differently in WCR susceptible larvae fed on control non-Bt maize compared with those fed on eCry3.1Ab-, mCry3A-, or Cry3Bb1-expressing maize seedlings. The metabolite annotation was confirmed with authentic standards, expected for the metabolites followed with (’) that were annotated based on their available identities (e.g., *m/z*, mass spectra). Asterisks (*) represent significant differences (p < 0.05) of metabolites in the susceptible insects fed on Bt maize compared with those fed on the non-Bt maize.

To further identify those metabolites associated with the differences in the OPLS-DA plot, pattern hunter analysis was conducted to identify metabolites that upregulated or downregulated in the susceptible insects when reared on three Bt maize lines compared to those fed on a non-Bt maize (Fig. 2). This analysis resulted in the identification of 16 upregulated metabolites and 16 downregulated metabolites that significantly were altered in the susceptible larvae fed on maize with and without the expression of Bt toxin (Table 3). The majority of upregulated metabolites in the susceptible insects reared on the Bt maize compared to those fed on the non-Bt maize were associated with lipid metabolism pathways, followed by nucleotide, amino acids and other metabolic pathways (Supplementary Table 2). The downregulated metabolites in these insects were involved in lipid metabolism pathways, cofactors and vitamins, amino acids, and other metabolism pathways (Supplementary Table 2).

**Table 3:** Metabolites with significantly altered expression in susceptible larvae fed on Bt-expressing maize lines (eCry3.1Ab, mCry3A, or Cry3Bb1) compared to those fed on a non-Bt maize control line.

Metabolite	eCry3.1Ab/non-Bt maize line		mCry3A/non-Bt maize line			Cry3Bb1/non-Bt maize line
	FC*	p-value	FC	p-value	FC	p-value
**Amino acids**						
2-Methylserine	1.56	0.060	1.04	0.080	1.95	**0.012**
Argininate’	1.18	0.126	1.10	0.401	1.27	**0.039**
*Fructosyllysine*	0.68	**0.024**	0.37	**< 0.001**	0.29	**< 0.001**
*Spermidine*	0.74	**0.031**	0.81	0.106	0.74	**0.033**
*o-Tyrosine*	0.80	0.130	0.70	**0.041**	0.79	0.140
**Nucleotide**						
N1-methylinosine	1.37	0.316	1.93	0.099	2.17	**0.049**
2′-O-methylcytidine	1.17	0.697	1.94	**0.035**	1.40	0.210
Cytidine 2′,3′-cyclic monophosphate	1.39	**0.017**	1.09	0.370	0.95	0.709
**Lipids**						
Arachidoyl-tetradecasphingadienine-phosphoethanolamine (d14:2/20:0)’	1.26	**0.031**	1.32	**0.014**	1.18	0.078
1-Oleoyl-2-linoleoyl-GPC (18:1/18:2)’	1.00	0.891	1.30	**0.050**	1.19	0.153
Sphinganine	1.36	0.120	2.05	**0.007**	1.71	**0.024**
Sphingomyelin (d18:1/18:1, d18:2/18:0)	1.35	**0.005**	1.42	**0.002**	1.15	0.095
Choline	1.37	**0.039**	1.12	0.325	1.06	0.556
Hexadecasphingosine (d16:1)’	1.29	0.351	1.75	**0.046**	1.35	0.249
Sphingosine	2.70	0.064	3.53	**0.036**	2.50	0.061
Campesterol	1.62	**0.009**	1.38	**0.041**	1.26	0.112
1-Palmitoyl-2-palmitoleoyl-GPC (16:0/16:1)’	0.99	0.991	1.43	**0.047**	1.31	0.094
*Eicosanedioate (C20-DC)*	0.58	0.058	0.60	0.053	0.53	**0.033**
*3-Hydroxysebacate*	0.61	**0.048**	0.67	0.090	0.72	0.155
*Dihomolinolenate (20:3n3 or 3n6)*	0.73	0.600	0.15	**0.008**	0.25	0.053
*Hexadecatrienoate (16:3n3)*	0.72	0.372	0.39	**0.031**	0.36	**0.039**
*1,2-Dilinolenoyl-GPC (18:3/18:3)’*	0.25	**0.016**	0.47	0.143	0.39	0.068
*β-sitosterol*	0.79	0.061	0.82	0.110	0.72	**0.017**
**Cofactors and vitamins**						
*Biotin*	0.79	0.385	0.38	**0.016**	0.54	0.063
*FAD*	0.51	**0.033**	0.63	0.092	0.76	0.215
*α-Tocopherol*	0.46	**0.003**	0.28	**< 0.001**	0.40	**0.001**
*Pyridoxal*	0.86	0.341	0.60	**0.014**	0.67	**0.044**
**Carbohydrates**						
Pentose acid*	0.32	**0.048**	0.42	0.093	0.40	0.064
**Energy**						
Malate	1.08	0.528	1.31	**0.029**	1.19	0.117
**Xenobiotics**						
Cyclic (AMP-GMP)	1.40	0.081	1.22	0.333	1.79	**0.012**
*2-Dimethylaminoethanol*	0.34	**0.002**	0.43	**0.004**	0.52	**0.014**
*Coniferin*	0.46	0.204	0.16	**0.014**	0.21	**0.016**

*Data are expressed as fold change (FC) and corresponding p-value. Fold changes were calculated by dividing relative concentrations of metabolites in the susceptible larvae fed on Bt maize lines expressing each of three available Cry3 proteins by that in those fed on the non-Bt maize line. Metabolites in *italic* represent higher levels in the susceptible larvae fed on the non-Bt maize isoline compared to Bt maize. All the other metabolites accumulated in larvae when reared on Bt maize compared with the non-Bt maize line. Metabolite identification was confirmed with authentic standards, expected for the metabolites followed with (’) that were annotated based on their available identities (e.g., *m/z*, mass spectra). Significant p values (< 0.05) were in bold.

### Comparative metabolite profiling in resistant strains fed on their respective Bt maize and non-Bt maize

There were 582 metabolites (~ 80% of the identified metabolite data set) detected in all samples of WCR resistant larvae fed on their respective Bt maize and non-Bt maize (Supplementary Table 3). The OPLS-DA was conducted to characterize differences in metabolite profiles between the resistant insects fed on their respective Bt maize (eCry3.1Ab, mCry3A, or Cry3Bb1) compared with those fed on the non-Bt maize. In the OPLS-DA plot, the profiles of the resistant insects for each resistant strain roughly shared a similar pattern regarding the orthogonal T score component and were similarly separated along the T score component (Fig. 1b).

**Figure 3:**
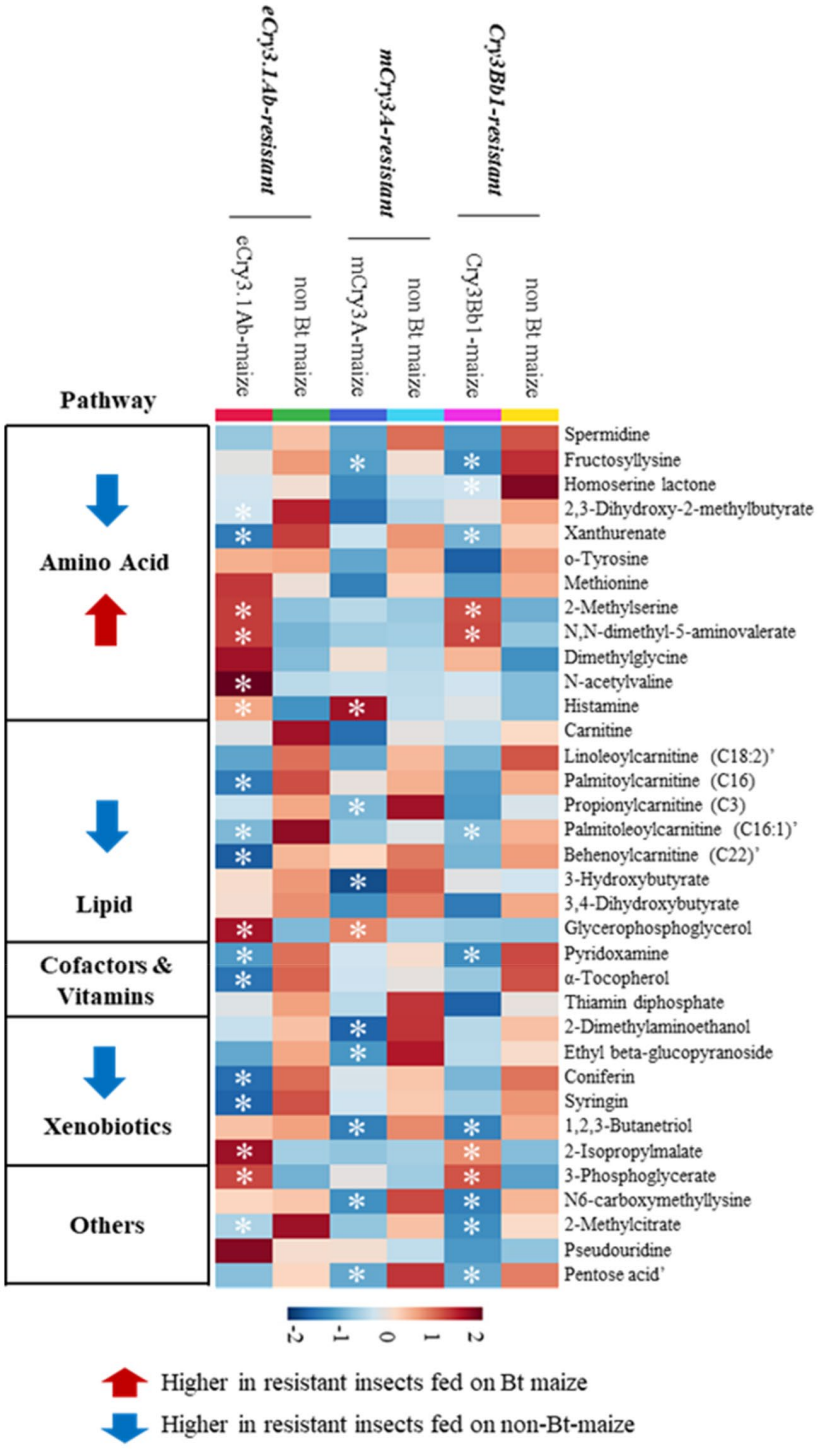
Heatmap for the identified metabolites expressed differently in WCR resistant larvae fed on control non-Bt maize compared with those fed on eCry3.1Ab-, mCry3A-, or Cry3Bb1-expressing maize seedlings. The metabolite annotation was confirmed with authentic standards, expected for the metabolites followed by (’) that were annotated based on their available identities (e.g., *m/z*, mass spectra). Asterisks (*) represent significant differences (p < 0.05) of metabolites in the resistant insect fed on their respective Bt maize with those fed on the non-Bt maize.

The pattern hunter analysis was performed to further detect those metabolites associated with the changes in the metabolite profiles of the resistant insects exclusively by Bt toxins (Fig. 3). This analysis resulted in the identification of 6 metabolites that were upregulated, and 19 metabolites that were downregulated in resistant insects fed on their respective Bt maize compared to those fed on non-Bt maize (Table 4). The majority of upregulated metabolites in the resistant insects fed on Bt maize compared to those fed on non-Bt maize were related to amino acid metabolism pathways (Supplementary Table 4). The downregulated metabolites in the resistant insects fed on Bt maize were classified into lipid, amino acid, cofactors and vitamins metabolism pathways, and other pathways (Supplementary Table 4). Six metabolites expressed differently were found in both susceptible and resistant larvae, including 2-methylserine, 2-dimethylaminoethanol, α-tocopherol, coniferin, fructosyllysine, and pentose acid.

**Table 4:** Metabolites with significantly altered expression in resistant larvae fed on three Bt-expressing maize lines compared with a non-Bt maize line.

Metabolite	eCry3.1Ab/non-Bt maize line		mCry3A/non-Bt maize line		Cry3Bb1/non-Bt maize line		
	FC*	p-value	FC	p-value	FC		p-value
**Amino acids**							
2-Methylserine	2.51	**0.001**	1.15	0.433	2.81		**< 0.001**
Histamine	5.04	**0.016**	2.67	**0.013**	1.82		0.250
*N*-Acetylvaline	2.36	**0.007**	1.02	0.828	1.36		0.252
*N*,*N*-dimethyl-5-aminovalerate	4.35	**< 0.001**	0.96	0.918	3.39		**0.002**
*Homoserine lactone*	0.93	0.721	0.78	0.163	0.67		**0.038**
*2,3-Dihydroxy-2-methylbutyrate*	0.61	**0.032**	0.61	0.031	0.81		0.275
*Fructosyllysine*	0.77	0.125	0.60	**0.010**	0.36		**< 0.001**
*Xanthurenate*	0.16	**< 0.001**	0.60	0.172	0.46		**0.049**
**Lipids**							
Glycerophosphoglycerol	3.63	**< 0.001**	2.10	**0.046**	1.03		0.946
*Behenoylcarnitine (C22)’*	0.42	**0.012**	0.83	0.600	0.61		0.170
*Palmitoylcarnitine (C16)*	0.44	**0.011**	0.86	0.700	0.60		0.101
*Palmitoleoylcarnitine (C16:1)’*	0.25	**0.005**	0.62	0.266	0.39		**0.045**
*Propionylcarnitine (C3)*	0.67	0.382	0.36	**0.017**	0.55		0.129
*3-Hydroxybutyrate*	0.91	0.884	0.60	**0.019**	1.02		0.888
**Cofactors and vitamins**							
*α-Tocopherol*	0.22	**0.012**	0.90	0.937	0.45		0.073
*Pyridoxamine*	0.58	**0.003**	0.91	0.490	0.53		**< 0.001**
**Carbohydrates**							
3-Phosphoglycerate	2.05	**0.003**	1.24	0.239	2.17		**0.002**
*N6-carboxymethyllysine*	0.97	0.921	0.57	**0.006**	0.62		**0.012**
*Pentose acid’*	0.68	0.161	0.45	**0.011**	0.51		**0.032**
**Energy**							
*2-Methylcitrate*	0.52	0.049	0.67	0.169	0.56		**0.040**
**Xenobiotics**							
2-Isopropylmalate	4.95	**0.002**	0.75	0.429	5.13		**< 0.001**
*Coniferin*	0.17	**0.002**	0.77	0.325	0.40		0.169
*Ethyl beta-glucopyranoside*	0.22	0.292	0.09	**0.012**	0.63		0.303
*Syringin*	0.19	**< 0.001**	0.76	0.521	0.55		0.101
*1,2,3-Butanetriol*	0.92	0.876	0.38	**0.014**	0.42		**0.040**
*2-Dimethylaminoethanol*	0.87	0.789	0.56	**0.010**	0.85		0.430

*Data are expressed as fold change (FC) and corresponding p-value. Fold changes were calculated by dividing relative concentrations of metabolites in the resistant larvae fed on their respective Bt maize line by that in those fed on the non-Bt maize line. Metabolites in italic represent higher levels in the resistant larvae fed on the non-Bt maize isoline compared to their respective Bt maize. All the other metabolites accumulated in the resistant larvae when reared on their respective Bt maize compared with the non-Bt maize line. Metabolite identification was confirmed with authentic standards, expected for the metabolites followed with (’) that were annotated based on their available identities (e.g., *m/z*, mass spectra). Significant p values (< 0.05) were in bold.

### Specific pathways in which the identified metabolites were involved

**Figure 4:**
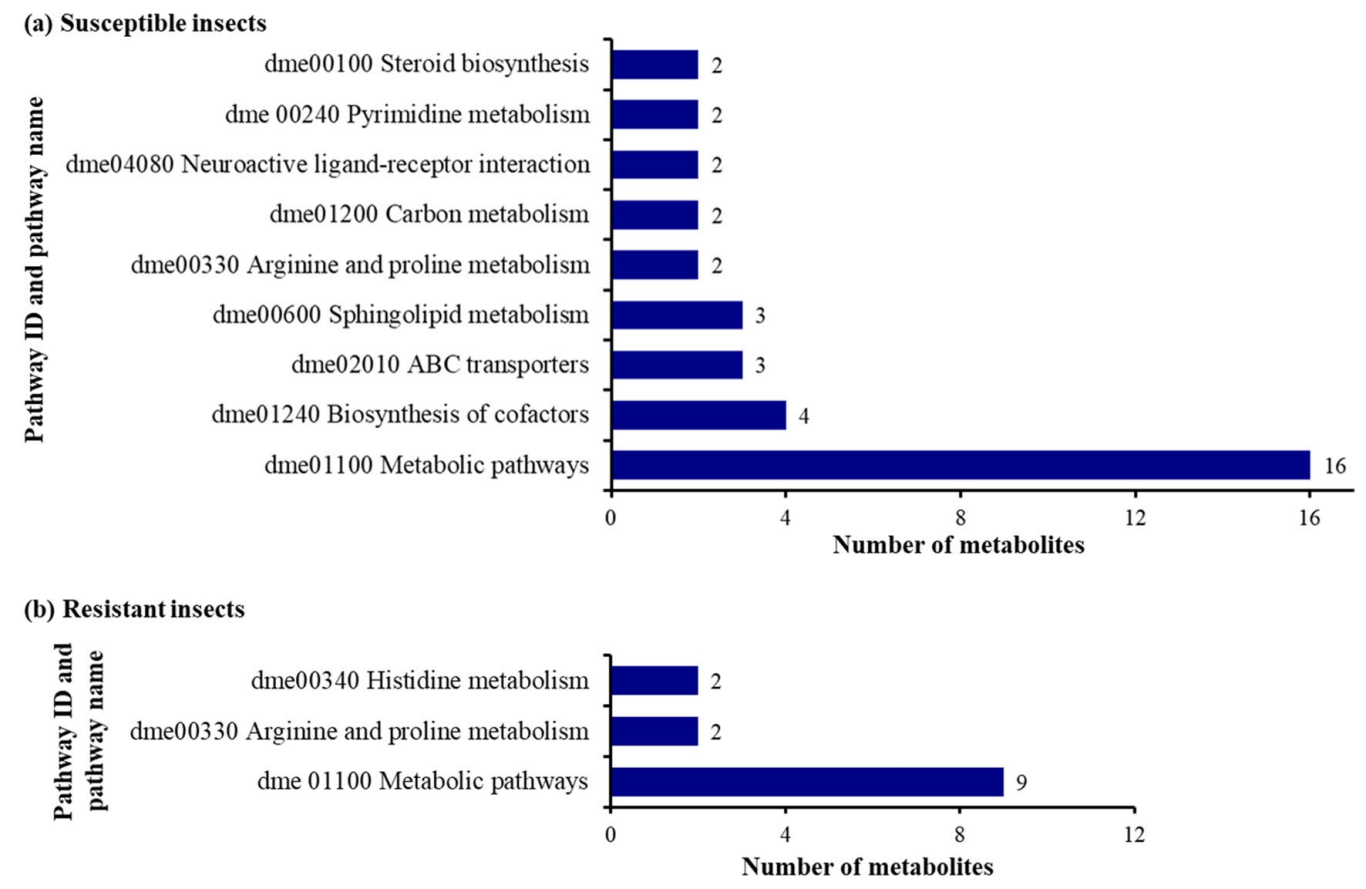
KEGG pathway analysis of differences in the identified metabolic pathways in susceptible (**a**), and resistant (**b**) western corn rootworm larvae fed on Bt-expressing maize seedlings (metabolite numbers ≥ 2).

The identified metabolites that expressed differently in susceptible and resistant insects were further located in the Kyoto Encyclopedia of Genes and Genomes (KEGG) database. In total, 16 metabolites out of 22 identified metabolites in susceptible insects were found to be involved in 29 pathways known in the KEGG *D. melanogaster* pathway library, whereas 9 metabolites out of 25 identified metabolites in resistant insects were involved in 17 pathways (Supplementary Tables 5, 6). Among these pathways, three pathways including biosynthesis of cofactors (dme01240), ABC transporters (dme02010), and sphingolipid metabolism (dme00600) were the pathways associated with most of the identified metabolites in the susceptible insects, while arginine and proline metabolism (dme00330) and histidine metabolism (dme00340) were found as the most related pathways for the resistant insects among the identified pathways (Fig. 4).

## Discussion

In the field, WCR has a history of evolving resistance to nearly every management tactic developed; thus, there is a logical concern that WCR will likely continue to develop resistance to current and new control methods targeting this pest. To mitigate future resistance development, it is critical to obtain a better understanding of resistance mechanisms in WCR. In the current study, we utilized the first global metabolomics analysis of WCR larvae to examine the shifts in larval WCR metabolome caused by the consumption of Cry3 toxins. This allowed the identification of the first WCR metabolome from susceptible and resistant strains to Bt maize expressing each of three currently available Cry3 proteins and a non-Bt maize. By systematically characterizing these metabolomic profiles, we determined a list of metabolites that may be involved in the intoxication of WCR larvae to Bt toxins, and identified possible pathways that may be associated with WCR recovery from Cry3 intoxication.

Our results revealed similarities and differences in metabolic profiles of susceptible WCR larvae fed on maize hybrids with and without the expression of Bt toxins. These inherent patterns were also observed when the susceptible larvae reared on Bt maize expressing different Cry3 proteins. The metabolomic analyses were performed on only susceptible larvae that had successfully developed to the 3rd instar. These larvae likely recovered from the ingestion of Bt toxins when feeding on Bt maize for 14 days, or were not exposed to Bt toxins when rearing on the non-Bt maize. Interestingly, we found a clear separation of the metabolic profiles of the susceptible larvae that was similarly distributed to one component but separated along another component in the multivariate analysis (Fig. 1a). These similar patterns could point out a possibility of similar metabolic pathways involved in the intoxication response of the susceptible insects to the three different Cry3 proteins. As suggested by cross-resistance studies^12^, the differences in toxicity levels between the different Cry3 proteins may contribute to differences seen in the metabolic profiles of the susceptible larvae. In the maize field, WCR adult emergence from eggs exposed to Bt transgenic maize roots expressing eCry3.1Ab, mCry3A, and Cry3Bb1 was 99.79%, 97.83%, and 98.49%, respectively^39–41^. Among the examined Bt maize, eCry3.1Ab expressing maize had the highest toxic effects on WCR larvae which is in agreement with the distribution of the associated metabolic profiles of the susceptible larvae fed on eCry3.1 expressing maize compared with those fed on other Bt maize and a non-Bt maize.

Interestingly, larvae resistant to eCry3.1Ab, mCry3A, and Cry3Bb1 expressing maize shared similarities in the shifts of their metabolic profiles when they were reared on their respective Bt maize compared to controls reared on a non-Bt maize. The changes in the metabolic profiles of the resistant larvae roughly shared the similar direction and distance in the multivariate analysis (Fig. 1b). This clear pattern may indicate metabolic responses against Bt intoxication shared in the resistant insects against the three Bt toxins. The Cry3Bb1-resistant strain was derived from a merger of multiple strains selected for resistance to Cry3Bb1. Meihls et al.^14^ selected for a Cry3Bb1-resistant strain using different techniques. Later, Meihls et al.^42^ selected multiple colonies for resistance to Cry3Bb1. Zukoff et al.^12^ evaluated several field-selected strains from Minnesota. A total of at least 8 Cry3Bb1-selected strains were merged at different times into the strain used in the current study. Cross-resistance between Cry3Bb1, mCry3A, and eCry3.1Ab has been clearly documented^12^. The Cry3Bb1-resistant insects may possess some level of cross-resistance between Cry3Bb1 and other Cry3 toxins (eCry3.1Ab and mCry3A), which may contribute to the similarities in the metabolic profiles between these insects and other Cry3-resistant insects. The differences in the metabolic profiles of the resistant insects may be due to the differences in genotype, Cry proteins, genetic background and toxic effects among the Bt maize evaluated^39–41^. Cry3Bb1-resistant insects were > 100-fold resistant, whereas eCry3.1Ab and mCry3A-resistant insects were > 13-fold and > 4-fold resistant, respectively^43^.

Our results indicated many metabolites significantly altered in susceptible and resistant larvae when feeding on Bt maize (Figs. 2, 3). These metabolites were classified into lipid, nucleotides, amino acids, cofactors and vitamins metabolism, xenobiotics, and other pathways. Among these metabolites, the metabolites in the xenobiotics pathways were plant secondary metabolites that may be not only undigested dietary materials present in the digestive system in larvae but also may play a role in pathways associated with the resistance in insects to xenobiotics^44^. Multiple metabolites were detected to be expressed differently in susceptible and resistant larvae, including 2-methylserine, 2-dimethylaminoethanol, α-tocopherol, coniferin, fructosyllysine, and pentose acid. Noticeably, susceptible and resistant larvae exhibited the depletion of α-tocopherol and vitamin B_6_ intermediates (pyridoxal and pyridoxamine) when feeding on Bt maize (Tables 3, 4). Tocopherol (vitamin E) is an essential lipid-soluble antioxidant encompassing eight forms: α-, β-, γ-, and δ-tocopherols, and the corresponding four tocotrienols. Depletion of tocopherols during WCR larval Bt intoxication may be related to their role as primary nonenzymatic antioxidants that prevent free radical peroxidative to unsaturated fatty acids within cellular membranes^45^ which occurs during Bt intoxication. Vitamin B_6_ is a cofactor required for many enzymatic reactions of amino acid synthesis and catabolism, fatty acid biosynthesis, glycogen mobilization, nucleotide synthesis, and neurotransmitter synthesis^46^. As an essential cofactor B_6_ involvement in these many pathways in response to Bt intoxication and repair-related metabolism could underlie the altered levels we report here for WCR larvae. Future studies examining whether the enzymatic activities and transcript levels within the WCR B_6_ biosynthetic pathways are altered by Bt intoxication could validate these metabolomic findings.

One of the proposed Bt mode(s) of action in insects is that Cry entomotoxic proteins are δ pore-forming proteins that disrupt the midgut epithelium, leading to death of the insect host by septicemia^17^. In agreement with this proposed mode of action, most of the identified metabolites in susceptible larvae were associated with biosynthesis of cofactors, ABC transporters and sphingolipid metabolism pathways (Fig. 4). Among eight subfamilies of ABC transporters identified in insects^47^, only ABCB1 was identified as a functional receptor of Cry3Aa in WCR, but not for insecticidal proteins against WCR including Gpp34Ab1/Tpp35Ab1, Cry6Aa1, and IPD072Aa^28^. Interestingly, among the multiple upregulated lipid metabolite pathways identified was the sphingolipid metabolism pathway that may be restricted to biological membranes^48^. These metabolites (e.g., sphingosine, sphinganine, sphingomyelin) are sufficiently amphipathic to diffuse between membranes and to flip between membrane leaflets^48^, which may be associated with the activity of Bt toxins binding to midgut border membranes.

For the resistant larvae, a few matches of the metabolites altered significantly in the resistant larvae exclusively by Bt maize in the KEGG pathway database for the insect model *Drosophila melanogaster*. These metabolites are mostly related to amino acid metabolism pathways (e.g., histidine metabolism, and arginine and proline metabolism). Only one metabolite (methionine) related to the ABC transporter metabolism pathway was upregulated in the resistant larvae when reared on Bt maize compared to those fed on non-Bt maize. Similar findings were reported by Porretta et al.^49^ that ABC transporters are not always involved in resistance to insecticides. Furthermore, the identified metabolites in susceptible and resistant larvae were found in 29 and 17 biochemical pathways respectively, which may suggest that several small changes may happen in both susceptible and resistant larvae that were likely recovery from Bt toxification when they successfully developed to 3rd instar. Further research could focus on different time intervals of the Bt toxification in which the larvae are in the process of detoxification and recovery would be needed to identify physiological effects and associated metabolism pathways of feeding on Bt versus non-transgenic maize by both susceptible and resistant larvae.

In summary, this is the first study to report the effects of dietary Cry3 entomotoxic proteins on the metabolome of susceptible and resistant western corn rootworm larvae. This resulted in a list of candidate metabolites accumulating in susceptible and resistant larvae that may be involved in WCR intoxication by each of three currently available Cry3 toxins targeting this pest. Multiple metabolic pathways associated with significantly altered metabolites in the susceptible and resistant insects were identified. Our results are consistent with recent similar toxicogenomics studies conducted on WCR larvae fed Bt proteins or Bt-expressing maize which reported elevated transcriptional levels of cellular pathways associated with stress, detoxification, free radical damage repair, apoptosis, autophagy, repair pathways, and ABC transporters (Cry3Bb1 and Gpp34/Tpp35Ab1^50^, eCry3.1Ab^33^, Cry3Bb1^32^, Gpp34Ab1, Tpp35Ab1 and Gpp34/TppAb1^51^). Validation of hypotheses generated with toxicometabolomic methods will require additional experimentation to determine which, if any, of the metabolites participate in WCR intoxication by Bt toxins. Supplementation of diets with selected compounds identified in this survey could alter larval responses to intoxication, i.e., compounds depleted during intoxication if provided in sufficient concentration might reverse susceptibility. Conversely addition of others might elevate resistance. Other contributors to the WCR metabolome are the WCR microbiome which is altered by the environment, life stage, and eCry3.1Ab feeding^26,52^. To characterize the identified biochemical pathways for a more complete understanding of the resistance of WCR to Bt proteins, other methods such as metabolic flux analysis could be applied^35^.

## Methods

### Plant materials

Three maize lines expressing individual Bt proteins, and a non-Bt maize line were utilized in this study. The maize lines expressing eCry3.1Ab protein (event 5307, material ID 12MG00345) and mCry3A protein (event MIR604, material ID 19MGO083079) were provided by Syngenta (Research Triangle Park, NC). The maize line expressing Cry3Bb1 protein (event MON88017, DKC61-88) was provided by Bayer Corporation (St. Louis, MO). The non-Bt maize line (Viking 60-01N) was purchased from Albert Lea Seed (Albert Lea, MN). The four maize lines were separately grown in plastic containers (0.7 L, 15.0 × 7.0 × 10.0-cm, The Glad Products Company, Oakland, CA), in a growth chamber maintained at 25 °C, ~ 80% RH, and a photoperiod of 14:10 (L:D) h, as described previously^53^ (see Supplementary Methods). After 3 days post-incubation in the growth chamber the plant containers were used for WCR rearing.

### Insects

Eggs from non-diapausing, susceptible and resistant WCR strains were obtained from the Plant Genetics Research Unit-USDA-ARS in Columbia, MO. The eCry3.1Ab and mCry3A-resistant strains were derived from the colonies that have > 45 generations of selection on eCry3.1Ab-expressing maize and mCry3A-expressing maize^54,55^. The eCry3.1Ab and mCry3A-resistant strains are > 13-fold and > 4-fold resistant compared to a susceptible strain, respectively^43^. The Cry3Bb1-resistant strain was derived from field strains with as much as 34.8-fold resistance to Cry3Bb1^12^. These feral strains were crossed with a non-diapausing strain and then selected in the lab on Cry3Bb1-expressing maize for ~ 20 generations. Finally, the resistant strains from Zukoff, et al.^12^ were merged into a single colony and then underwent an additional 6 generations of selection on Cry3Bb1-expressing maize^12^. This strain is > 100-fold resistant compared to a susceptible strain^43,56^. The susceptible strain was originally purchased from Crop Characteristics (non-diapausing WCR; Farmington, MN) and was maintained on a non-Bt maize line (Viking 60-01N) for multiple generations.

### Experimental design

WCR larvae from resistant strains were reared on their respective maize lines and a non-Bt maize line, whereas WCR larvae from a susceptible strain were allowed to feed on all the four maize lines evaluated (Table 1). Only WCR larvae that developed to the 3rd instar after 14 days of feeding on maize were collected for metabolic profiling analysis. Each treatment consisted of 50 WCR larvae (~ 500 mg per treatment) fed on a Bt maize line, while WCR larvae reared on a non-Bt maize were included as the controls. Three replicates per treatment were used for the metabolite extraction and analyses.

For insect rearing, eggs were obtained in Petri dishes consisting of eggs and soil. These eggs were incubated at 25 °C in complete darkness in an incubator (Percival, Perry, IA) until approximately 5% of the eggs were hatched. Subsequently, the eggs were washed from soil with tap water and were collected in a 1 ml disposable pipette (13-711-9a, Fisher Scientific, Pittsburg, PA). The eggs were dispensed from the pipette onto a coffee filter paper (Pure Brew, Rockline Industries, Sheboygan, WI). The coffee filter was then placed inside a 11.7 × 7.62 × 9.6-cm container with a lid (LG8RB-0090 & DM16R-0090, Solo Cup Company, Lake Forest, IL) as described previously^57,58^. The egg containers were incubated at 25 °C in darkness and larvae that hatched within 24 h were used for insect assays. Sixty WCR neonates that hatched within 24 h were infested to each plant container using a fine paintbrush. The containers were then placed in the growth chamber maintained at 25 °C, ~ 60% RH, and a photoperiod of 14:10 (L:D) h for 14 days. The containers were checked daily and were watered as needed to ensure that the soil was moist. Since percent adult emergence from eggs of WCR on Bt transgenic maize roots expressing eCry3.1Ab, mCry3A, and Cry3Bb1 on WCR larvae was 0.21%, 2.17%, and 1.51%, respectively in the field^39–41^, several plant containers of each maize line were prepared for WCR rearing to obtain sufficient samples for the metabolite extraction. All the containers were prepared within 1 week. At day 14 post infestation, WCR larvae that survived to the 3rd instar were removed from soil and maize roots in the plant containers and were transferred to a moist filter paper using forceps. WCR larvae were then collected into 2 mL vials (50 larvae per vial or ~ 500 mg per vial) and were immediately flash-frozen in liquid nitrogen. The WCR samples were stored in a − 80 °C freezer until the metabolite extractions.

### Metabolite extraction

The WCR samples were extracted and analyzed by Metabolon, Inc. (Morrisville, NC)^59^. The samples were prepared for the extraction using an automated MicroLab STAR^®^ system (Hamilton Company, Reno, NV). Prior to the first step in the extraction process, several recovery standards (i.e., dl-2-fluorophenylglycine, tridecanoic acid, d6-cholesterol, and 4-chlorophenylalanine) were added to the samples. The samples were then extracted with methanol under vigorous shaking for 2 min (Glen Mills Geno/Grinder 2000, SPEX CertiPrep, Metuchen, NJ) to precipitate protein and dissociate small molecules bound to protein or trapped in the precipitated protein matrix, followed by centrifugation to recover chemically diverse metabolites. The resulting extracts were placed briefly on a TurboVap^®^ (Zymark, Clackamas, OR) to remove the organic solvent. The sample extracts were stored overnight under nitrogen before preparation for metabolite analysis.

### Ultrahigh performance liquid chromatography-tandem mass spectroscopy (UPLC-MS/MS) to identify metabolite profiles

The sample extracts were analyzed by a Waters ACQUITY ultra-performance liquid chromatography (UPLC, Waters Corporation, Milford, MA) and a Thermo Scientific Q-Exactive high resolution/accurate mass spectrometer (Thermo Scientific, Waltham, MA) interfaced with a heated electrospray ionization (HESI-II) source and Orbitrap mass analyzer operated at 35,000 mass resolution. Initially, each extract was divided into five fractions: two for analysis by two separate reverse phases (RP)/UPLC-MS/MS methods using positive ion mode electrospray ionization (ESI), one for analysis by RP/UPLC-MS/MS using negative ion mode ESI, one for analysis by hydrophilic interaction liquid chromatography (HILIC)/UPLC-MS/MS using negative ion mode ESI, and one reserved for backup. The sample extract was dried and then reconstituted in solvents compatible with each of the four methods. Each reconstitution solvent contained a series of standards (i.e., d7-glucose, d3-leucine, d8-phenylalanine, d5-tryptophan, d5-hippuric acid, Br-phenylalanine, d5-indole acetic acid, amitriptyline, and d9-progesterone) at fixed concentrations to ensure injection and chromatographic consistency. The resulting aliquots were analyzed as described in the Supplementary Methods.

### Data extraction and compound identification

Raw data were extracted, peak-identified, and processed using Metabolon’s platform that runs on high-performance application servers and fiber-channel storage arrays in clusters to provide active failover and load-balancing. Compounds were identified by comparison to Metabolon’s library consisting of over 4500 commercially available authenticated standard compounds with the retention time/index (RI), mass to charge ratio (*m/z)*, and chromatographic data (including MS/MS spectral data). Additionally, the annotation of the compound was based on mass spectral entries with structurally unnamed biochemicals which have been identified by virtue of their recurrent nature (both chromatographic and mass spectral). Biochemical identifications were further based on three criteria: retention index within a narrow RI window of the proposed identification, accurate mass match to the library ± 10 ppm, and the MS/MS forward and reverse scores. MS/MS scores are based on a comparison of the ions present in the experimental spectrum to ions present in the library entry spectrum. If there were similarities between these molecules based on one of these factors, the use of all three data points was utilized to distinguish and differentiate biochemicals.

### Data processing and analysis

To determine differences in the metabolic profiles of susceptible and resistant larvae reared on maize lines expressing different Bt toxins and without Bt toxins, orthogonal partial least squares—discriminant analyses (OPLS-DA) were performed using a web-based tool MetaboAnalyst 5.0 (https://www.metaboanalyst.ca)^60^ (see Supplementary Methods).

To detect metabolites that may be involved in WCR defense to Bt toxins, we identified metabolites that were downregulated when WCR larvae from both resistant and susceptible strains fed on non-Bt maize but upregulated in those reared on Bt maize and vice versa. The analysis was performed using the pattern hunter algorithm in the MetaboAnalyst platform^61^ (see Supplementary Methods). The metabolites that had the Pearson correlation > 0.5 or < − 0.5 were selected for further analyses.

To determine the difference in the metabolites that may be involved in WCR defense against Bt toxins, the metabolites identified from the pattern hunter algorithm were analyzed with analysis of variance (ANOVA) using PROC MIXED in SAS 9.4 (SAS Institute, Cary, NC). WCR larvae fed on maize was the fixed effect and replication was the random variable. Differences between the treatments were determined using Fisher’s least significant difference (LSD) at p < 0.05. The relative concentrations (intensity) of the identified metabolites were logarithm transformed prior to the analysis to meet assumptions of normality and homoscedasticity.

To identify the pathways possibly associated to WCR responses to Bt toxins, the metabolites altered significantly in susceptible and resistant larvae were submitted to the KEGG pathway database (http://www.kegg.jp/kegg/pathway.html)^62^. *Drosophila melanogaster* was used as an insect model for pathway identification.

## Supplementary Information

Supplementary Information.

Supplementary Tables.

## Data Availability

All pertinent data are found in the figures and tables. Requests for data and additional information should be submitted to the corresponding author.
