# Supplementary material for: Toxicometabolomic profiling of resistant and susceptible western corn rootworm larvae feeding on Bt maize seedlings
Source: Sci Rep. 2022 Jul 8;12:11639. doi: 10.1038/s41598-022-15895-z (PMC9270432; doi:10.1038/s41598-022-15895-z)
Supplement: Supplementary file 1 — Supplementary Information. [file 41598_2022_15895_MOESM1_ESM.docx]

**Toxicometabolomic profiling of resistant and susceptible western corn rootworm larvae feeding on Bt maize seedlings**

Man P. Huynh ^1,2*^, Bruce E. Hibbard ^3^, Khanh-Van Ho ^4,5,6^, and Kent S. Shelby ^7*^

^1^ Division of Plant Science & Technology, University of Missouri, Columbia, Missouri, USA

^2^ Department of Plant Protection, Can Tho University, Can Tho, Vietnam

^3^ Plant Genetics Research Unit, USDA-Agricultural Research Service, Columbia, Missouri, USA

^4^ Department of Chemistry, University of Missouri, Columbia, Missouri, USA

^5^ Molecular Imaging and Theranostics Center, University of Missouri, Columbia, Missouri, USA

^6^ Department of Food Technology, Can Tho University, Can Tho, Vietnam

^7^ Biological Control of Insects Research Laboratory, USDA-Agricultural Research Service, Columbia, Missouri, USA

* Corresponding authors: manhuynh@missouri.edu; kent.shelby@usda.gov

**Supplementary Methods**

**Plant materials.**  The four maize lines were separately grown in plastic containers (0.7 L, 15.0 × 7.0 × 10.0-cm, The Glad Products Company, Oakland, CA), in a growth chamber maintained at 25 °C, ~80% RH, and a photoperiod of 14:10 (L:D) h, as described previously[^1^](#_ENREF_1). To prepare the plant containers, 20 ml of tap water was added to the plastic containers, followed by an addition of 60 maize seeds. The seeds were then covered evenly by 100 ml of growth medium consisting of a 2:1 mixture by volume of autoclaved soil:Pro-Mix BX potting medium (Premier Horticulture Inc., Quakertown, PA) and 80 ml of tap water, respectively.

**Ultrahigh performance liquid chromatography-tandem mass spectroscopy (UPLC-MS/MS)** **to identify metabolite profiles.** The first aliquot was analyzed using acidic positive ion conditions, chromatographically optimized for more hydrophilic compounds. In this method, the extract was gradient-eluted from a C18 column (Waters UPLC BEH C18-2.1 × 100 mm, 1.7 µm particle size) using water and methanol, containing 0.05% perfluoropentanoic acid (PFPA) and 0.1% formic acid (FA). A second aliquot was also analyzed using acidic positive ion conditions but was chromatographically optimized for more hydrophobic compounds. In this method, the extract was gradient eluted from the aforementioned C18 column using methanol, acetonitrile, water, 0.05% PFPA and 0.01% FA, and was operated at an overall higher organic content. A third aliquot was analyzed using basic negative ion optimized conditions using a separate dedicated C18 column. The basic extracts were gradient-eluted from the column using methanol and water, however with 6.5 mM ammonium bicarbonate at pH of 8. The fourth aliquot was analyzed via negative ionization following elution from a HILIC column (Waters UPLC BEH Amide 2.1 × 150 mm, 1.7 µm) using a gradient consisting of water and acetonitrile with 10 mM ammonium formate, pH of 10.8. The MS analysis alternated between MS and data-dependent MS^n^ scans using dynamic exclusion. The scan range varied slightly between methods, but covered approximately 70 - 1000 m/z. Raw data files were archived and extracted as described below.

**Data processing and analysis.**

**Orthogonal partial least squares - discriminant analyses (OPLS-DA).** The data that included the identified compounds detected in WCR larvae with their relative concentrations (intensity) were uploaded to the MetaboAnalyst platform. The metabolite data were processed to remove missing data to select only metabolites detected in all replicates of the samples. The data were logarithm transformed and then were normalized by median. The resulting data were mean-centered and divided by the standard deviation of each variable for data scaling. The OPLS-DA was examined to identify separated patterns of the sample sets using the transformed data.

**Pattern hunter analyses.** Defined patterns specified for a series of numbers corresponding to the expression levels in corresponding groups were analyzed with Pearson correlation coefficient. In fact, the pattern “2^+^ – 2^+^ – 2^+^ – 1” corresponding to the susceptible insects “susceptible larvae fed on eCry3.1Ab-expressing maize – susceptible larvae fed on mCry3A-expressing maize – susceptible larvae fed on Cry3Bb1-expressing maize – susceptible larvae fed on non Bt maize” was analyzed to identify the metabolites downregulated (positive correlation) or upregulated (negative correlation) in the susceptible insects exclusively by Bt toxins. Additionally, the pattern “2^+^ – 1 – 2^+^ – 1 – 2^+^ – 1” corresponding to the resistant insects “eCry3.1Ab-resistant larvae fed on eCry3.1Ab-expressing maize - eCry3.1Ab-resistant larvae fed on non Bt maize – mCry3A-resistant larvae fed on mCry3A-expressing maize – mCry3A-resistant larvae fed on non Bt maize – Cry3Bb1-resistant larvae fed on Cry3Bb1-expressing maize – Cry3Bb1-resistant larvae fed on Cry3Bb1-expressing maize” was performed to determine the metabolites downregulated (positive correlation) or upregulated (negative correlation) in the resistant insects exclusively by Bt toxins.

**Supplementary References**

1 Pereira, A. E. *et al.* Baseline susceptibility of a laboratory strain of northern corn rootworm, *Diabrotica barberi* (Coleoptera: Chrysomelidae) to *Bacillus thuringiensis* traits in seedling, single plant, and diet-toxicity assays. *J. Econ. Entomol.* **113**, 1955-1962 (2020).
